# Satisfaction with medication in coronary disease treatment: psychometrics of the Treatment Satisfaction Questionnaire for Medication

**DOI:** 10.1590/1518-8345.0745.2705

**Published:** 2016-06-07

**Authors:** Ana Carolina Sauer Liberato, Roberta Cunha Matheus Rodrigues, Thaís Moreira São-João, Neusa Maria Costa Alexandre, Maria Cecília Bueno Jayme Gallani

**Affiliations:** 1 Doctoral student, University of Washington, Seattle, USA.; 2 Associate Professor, Faculdade de Enfermagem, Universidade Estadual de Campinas, Campinas, SP, Brazil.; 3 Professor, Faculdade de Enfermagem, Universidade Estadual de Campinas, Campinas, SP, Brazil.; 4 Full Professor, Faculty of Nursing, Laval University, Quebec, Canada.

**Keywords:** Nursing, Patient Satisfaction, Medication Adherence, Coronary Disease, Psychometrics

## Abstract

**Objective:**

to psychometrically test the Brazilian version of the Treatment Satisfaction Questionnaire for Medication - TSQM (version 1.4), regarding ceiling and floor effect, practicability, acceptability, reliability and validity.

**Methods:**

participants with coronary heart disease (n=190) were recruited from an outpatient cardiology clinic at a university hospital in Southeastern Brazil and interviewed to evaluate their satisfaction with medication using the TSQM (version 1.4) and adherence using the Morisky Self-Reported Measure of Medication Adherence Scale and proportion of adherence. The Ceiling and Floor effect were analyzed considering the 15% worst and best possible TSQM scores; Practicability was assessed by time spent during TSQM interviews; Acceptability by proportion of unanswered items and participants who answered all items; Reliability through the Cronbach's alpha coefficient and Validity through the convergent construct validity between the TSQM and the adherence measures.

**Results:**

TSQM was easily applied. Ceiling effect was found in the side effects domain and floor effect in the side effects and global satisfaction domains. Evidence of reliability was close to satisfied in all domains. The convergent construct validity was partially supported.

**Conclusions:**

the Brazilian TSQM presents evidence of acceptability and practicability, although its validity was weakly supported and adequate internal consistency was observed for one domain.

## Introduction

*Satisfaction* is a patient reported outcome that considers the patients' evaluation of aspects of the medical treatment and health care systems^1^. The interest in these types of measures has increased over the past decades, since patients have come to be considered as "consumers" and not just as passive receivers of health services^2^. 

While patient satisfaction with treatment includes the assessment of doctor-patient interaction, as well as other concomitant therapies, patient satisfaction with drug therapy is related only to medications^2^. 

The satisfaction with medication can be defined as the patient's evaluation on the process of taking the medications and the associated results of its use^2^. Patients' satisfaction with their medication has been demonstrated to predict the continuation on drug treatment, as well as adherence to correct and consistent use of drug therapy over time^3^. 

Prior studies in cardiology showed that patients who were more than 80% adherent to the protocol prescription had better clinical outcomes than those who were nonadherent^4^. Among the factors that possibly influence the medication adherence construct, the patient satisfaction with drug therapy stands out^2^.

Adherence to long-term drug therapy has proven to be essential for prevention and control of Coronary Heart Disease (CHD). Studies suggest that adherence to drug treatment over time results in the reduction of new ischemic events^5^ and optimization of survival and health-related quality of life of patients with CHD^6^. Moreover, the international literature indicates poor adherence to the pharmacological treatment among these patients^4^. 

Considering the clinical relevance of knowing patients' satisfaction with treatment, researchers have developed a wide-ranging questionnaire, aimed at measuring satisfaction with medication. The Treatment Satisfaction Questionnaire for Medication version 1.4 (TSQM) is a generic and broad-based outcome of patient satisfaction with medication, which has been validated among individuals with different chronic disease conditions^3^. 

The validity and reliability of the TSQM have been demonstrated in English, Spanish, Arabic and French, providing a robust tool to assess patients' satisfaction with the drug treatment to treat a variety of disease conditions^3^ ^,^ ^7^ ^-^ ^9^. Until this moment, the TSQM (version 1.4) had not been tested or validated for patients with CHD. 

No other psychometrically validated treatment satisfaction with medication measures are available for use with patients with Brazilian Portuguese as their native language. In addition, it has been demonstrated that patient satisfaction with medication predicts medication adherence^3^, so that satisfied patients are expected to be more adherent to prescribed therapeutic regimens^2^. It is also important to highlight that, if nurses evaluate patient satisfaction, it will be possible to prevent dissatisfaction and, consequently, non-adherence, making possible disease control and improvements in their quality of life^6^.

Given the importance of providing a reliable and valid tool for measuring patient satisfaction in relation to drug therapy for the Brazilian scientific community, the aim of this study was to psychometrically test the Brazilian version of the TSQM (version 1.4) when applied to participants with CHD, specifically verifying the practicability, acceptability, reliability, ceiling and floor effects and construct validity of the Brazilian version of the TSQM (version 1.4).

## Methods

### Study design and participants

A methodological study design was used to reach the research goals. The study was conducted at an outpatient clinic specialized in cardiology, at a teaching hospital in a large urban center in Southeastern Brazil. Participants were recruited by convenience, on the day of their regular consultation with the cardiologist, when they were invited to participate in the research. Their agreement was formalized by signing the consent form (consent rate was 100%). The sample comprised participants with CHD aged over 18 years, with unstable angina and/or myocardial infarction. Additional inclusion criteria were: to be able to effectively communicate verbally and be a current user of at least two life-saving drugs for treatment of CHD for a full month prior to study enrollment. 

### Sample size

The sample size was determined based on preliminary data, considering the following: Pearson correlation coefficient r=0.60 expected between measures of self-reported adherence and satisfaction with drug use, precision of 0.3 and a significance level of 5% (α=0.05)^10^. A sample size of at least 86 participants was determined. Data from 190 participants were gathered during the data collection period.

### Data collection

One researcher collected the data between June 2010 and May 2011 through structured interviews and consultation of the medical chart.

Sociodemographic and clinical data were gathered using a validated questionnaire^11^. Subsequently, a structured interview was conducted in order to measure satisfaction with the drug therapy by the application of the TSQM (version 1.4). Medication adherence was measured by the Morisky Self-Reported Measure of Medication Adherence and by quantifying the proportion of adherence.

### Data analysis

Data were analyzed using the SPSS Statistics - version 20 (IBM software), for the following analyses:

*Ceiling and Floor effects:* The participants' 15% worst results of the scale were considered the floor effect and, for the 15% best possible results, the ceiling effect^12^ was evaluated. 

*Practicability:* Assessed by the time spent during TSQM interviews.

*Acceptability:* Assessed by the proportion of unanswered items and participants who answered all items.

*Reliability:* The internal consistency was evaluated through Cronbach's alpha, and consistency for Cronbach's alpha was considered to be higher than 0.70^13^. 

*Validity:* The Pearson correlation coefficient was applied to test the convergent construct validity between the scores of the Brazilian TSQM and the score on the Brazilian *Morisky Self-Reported Measure of Medication Adherence* and the proportion of drug adherence. Significant correlations close to 0.30 were considered satisfactory, despite a small practical value; coefficients from 0.30 up to 0.50 were considered moderate and coefficients superior to 0.50 were considered strong^14^. A significance level of 0.05 (p-value) was adopted.

### Questionnaires

*Treatment Satisfaction Questionnaire for Medication - TSQM (version 1.4)*

The TSQM (version 1.4) is a questionnaire that measures the satisfaction with the drug therapy, considering the last two or three weeks or since the last time the patient took the medication. It is suggested that TSQM should be applied as a self-reported measure^3^ but, in this study, the TSQM was applied as part of an interview for the participants to easily comprehend the questions. There are 14 questions, distributed over 4 domains: effectiveness, side effects, convenience and global satisfaction. Responses were measured through a Likert-type scale of 5 or 7 points, and one dichotomous response (question 4). The TSQM (version 1.4) domain scores were calculated as recommended by the instrument's authors, which is described in detail elsewhere^15^ ^-^ ^16^. The score ranges from 0 to 100 in each domain and, the higher the score, the greater the patient satisfaction with medication^3^. 

Treatment satisfaction was assessed using the Brazilian version of the TSQM (version 1.4), and the copyright was obtained from Quintiles Strategic Research Services. The version 1.4 was translated into Brazilian Portuguese by qualified translators of the Center on Outcomes Research and Education of the United States (CORE)^17^. The method requires two forward translations into the Portuguese language by native speakers. Reconciled versions of the two forward translations were done by a third independent translator who was a native Portuguese speaker, a back-translation of the reconciled version by a native English speaker fluent in Portuguese and three reviews by native speaking linguists or health-related researchers. After the translation, the TSQM Brazilian version was linguistically validated in a Brazilian sample. These data is not published, however, it is available on a certificate offered by Quintiles. 

### Morisky Self-Reported Measure of Medication Adherence Scale

Consists of a short questionnaire aimed at assessing factors related to adherence, developed by Morisky and collaborators^18^. It consists of four questions with the answers measured in a Likert-type scale ranging from 1 to 4^19^. The sum of responses of the four items generates a score that ranges from four to eighteen, considering that the lower the score, the more favorable for adherence to drug use.

### Proportion of medication adherence

This instrument identifies and quantifies the drugs in current use, in order to provide the proportion (%) of drug adherence. It comprises four variables: 1) Description of the name and dosage of all drugs prescribed; 2) Description of the form of use of each drug according to the prescribed dose during the last 24 hours, 3) during the last week and 4) during the last month^19^.

*Adherence* was calculated based on missed doses over the last month, reported by the patient using the following calculation: [(prescribed doses - missed doses) / prescribed dose] x 100%^20^. Participants were considered adherent if a percentage of consumption of prescribed drugs higher or equal to 80% was obtained^4^. For individuals who used more than one drug, the proportion of the use was calculated for each drug and then the average percentage of adherence of all medications was calculated.

### Ethical considerations

The investigation conforms to the principles outlined in the Declaration of Helsinki and complies with resolution 196/96. The Faculty of Medical Science Ethics Committee from University of Campinas approved this study (Protocol: 2010/07332-0).

## Results

### Sociodemographic and clinical data

The study sample consisted of 190 participants. The sample was composed mainly of men (63.2%) with a mean age of 60.2 (SD 10) years, whites 135 (71.1%), with companion 135 (71.1%), professionally active 59 (31%), with 5.4 (SD 4) years of study (Table 1).

**Table 1:** Sociodemographic characteristics of participants with coronary heart disease (n=190), Campinas, SP, Brazil, 2010-2011

Sociodemographic Variables	n (%)	Mean (SD^*)^	Range	Median
Gender				
Male	120 (63.2)			
Education (years)		5.4 (4)	0-20	4
Age (years)		60.2 (10)	28-87	59.5
Marital Status				
With companion	135 (71.1)			
Race				
Caucasoid	135 (71.1)			
Professional Status				
Active	59 (31)			
Inactive	109 (57.3)			
Housewife	22 (11.6)			
Monthly income (US$)^†^				
Family		921.4 (665.1)	0-3,447.1	734.5

*SD = Standard Deviation; †Dollar exchange rate: 1.77 on 07/12/2011.

The majority 112 (58.9%) of the participants was diagnosed with Acute Coronary Syndrome with ST-segment elevation. Most presented dyslipidemia 184 (96.8%), hypertension 172 (90.5%) and diabetes 85 (44.7%). A large percentage 125 (65.8%) was composed of smokers. For many, chest pain 103 (54.2%), dyspnea 93 (48.9%) and lipothymia 88 (46.3%) were experienced over the last month. The mean number of symptoms in the sample was 2.4 (SD 1.5). Most participants 126 (66.3%) had been submitted to clinical treatment and intervention (Myocardial Revascularization and/or percutaneous angioplasty) (Table 2).

**Table 2:** Clinical characteristics of participants with coronary heart disease (n=190), Campinas, SP, Brazil, 2010-2011

Clinical Variables	n (%)	Mean (SD^*)^	Range	Median
Acute Coronary Syndrome (ACS - n=189)				
ST-segment elevation	112 (58.9)			
Unstable angina	40 (21.1)			
Non ST-segment elevation	37 (19.5)			
Years since the last ischemic event		8.3 (4.9)	3-27	6
Number of previous MI		1.4 (1.4)	0-10	1
Associated conditions				
Dyslipidemia	184 (96.8)			
Hypertension	172 (90.5)			
Diabetes Mellitus	85 (44.7)			
Number of clinical conditions		4 (1.7)	1-15	4
Smoking (current and past)	125 (65.8)			
Symptoms over the last month				
Chest pain	103 (54.2)			
Dyspnea	93 (48.9)			
Lipothymia	88 (46.3)			
Number of symptoms over the last month		2.4 (1.5)	0-6	2
Treatment of ACS				
Clinical	61 (32.1)			
Clinical and chirurgical	126 (66.3)			

*SD: Standard Deviation; †Left Ventricular Ejection Fraction, ‡LVEF≤58.0%.

### Practicability, acceptability and ceiling/floor effects

Regarding the acceptability, only 4 items were not answered among the participants enrolled (Question 2 - 1 time not answered; question 11 - 3 times not answered; question 12 - 7 times not answered; question 13 - 2 times not answered). As for the practicability, the TSQM demonstrated average application time of 4.6 (SD 2) minutes. Descriptive data of the measures of the TSQM, the Morisky Self-Reported Measure of Medication Adherence Scale and the proportion of adherence are presented in Table 3. 

**Table 3:** Descriptive analyses of medication adherence and satisfaction. Campinas, SP, Brazil, 2010-2011

Variables	n	%	Mean (SD*)	Range	Median
Satisfaction Measure					
TSQM (version 1.4)					
Effectiveness	189		67.7 (8)	27.8-88.9	66.7
Side Effects	190		93.5 (16.9)	12.5-100	100
Convenience	190		66.3 (9.5)	33.3-100	66.7
Global satisfaction	190		69.2 (12.6)	14.3-100	71.4
Adherence Measures					
Morisky Self-Reported Scale	190		5.8 (2.2)	4-15	5
Adherence proportion					
Life-savings drug adherence^†^					
Beta-blockers	143		94.4 (16)	0-100	100
Adherent		88.8			
Antiplatelet	170		92.5 (24.1)	0-100	100
Adherent		90.6			
Statins	127		95.5 (16.3)	0-100	100
Adherent		91.8			
Life-savings drugs (associated)^‡^	190		94.2 (13)	0-100	100
Adherent		87.9			
Drugs for symptom relief^§^					
Nitrate	45		88.4 (25.5)	0-100	100
Adherent		84.4			
Diuretic	90		96.7 (12.4)	13-100	100
Adherent		93.3			
Digitalis	7		36.1 (47.7)	0-100	0
Adherent		28.6			
Drugs for symptom relief (associated)^§^	190		93.1 (14.1)	0-100	100
Adherent		86.3			

* SD: Standard Deviation; ^†^Individuals who presented adherence ≥80% were considered adherent; ^‡^Drug therapy related to the reduction of morbidity and mortality in coronary heart disease - life-saving therapy - converting enzyme inhibitor (ACEI) or angiotensin receptor antagonists (ARBs), beta-blockers, antiplatelet agents and statins; ^§^Drugs used for symptoms relief in coronary heart disease: digoxin, diuretics and nitrates.

Considering that the scores of the Brazilian TSQM ranged from 0 up to 100 in each domain and that, the higher the score, the greater the satisfaction with the drug therapy. Higher scores were found in the side effects 93.5 (SD 16.9) domain and lower scores were reported in the convenience 66.3 (SD 9.5), effectiveness 67.7 (SD 8) and global satisfaction 69.2 (SD 2.6) domains (Table 3). Regarding self-reported measures of adherence, participants with a proportion of adherence larger than 80% were consider adherent; for the life-saving medications, the adherence was 94.2 (SD 13) and, for symptoms-relief medication, the score was 93.1 (SD 12.1). Analyses of the Morisky Self-Reported Measure of Medication Adherence also demonstrated the presence of factors related to the adherence in the sample with 5.8 (SD 2.2). 

The analyses of the ceiling and floor effects provide evidence that there was ceiling effect in the side effects (90.5%), convenience (14.2%), effectiveness (15.8%) and global satisfaction (25.8%) domains. The floor effect was discreetly observed in the side effects (0.5%) and global satisfaction (1.1%) domains.

### Reliability Analyses

Evidences of internal consistency were observed in TSQM (version 1.4), as the Cronbach's alpha (α) was satisfactory for side effects α=0.71 domain and close to satisfactory for the effectiveness α=0.69; convenience α=0.67 and global satisfaction α=0.69 domains. 

### Validity

Contrary to the assumptions previously established, significant correlations were small or not found between the scores of TSQM (version 1.4) and the scores on the Brazilian version of the Morisky Self-Reported Measure of Medication Adherence and the proportion of adherence (Table 4). 

**Table 4:** Pearson's correlation coefficients between TSQM (version 1.4) and adherence measures. Campinas, SP, Brazil, 2010-2011

*r	Adherence^†^ - Symptoms relief	Adherence^†^- Life-saving	Morisky	TSQM - *Effectiveness*	TSQM - *Side Effects*	TSQM - *Convenience*	TSQM - *Global Satisfaction*
Adherence^†^ - Symptoms relief	1.0						
Adherence^†^ - Life-saving	0.82^‡^	1.0					
Morisky	-0.24^‡^	- 0.26^‡^	1.0				
TSQM - *Effectiveness*	0.02	0.06	-0.07	1.0			
TSQM - Side Effects	0.16^§^	0.04	-0.18^§^	0.11	1.0		
TSQM - *Convenience*	-0.03	0.05	-0.09	0.19^‡^	0.02	1.0	
TSQM - *Global Satisfaction*	-0.04	0.03	-0.16^‡^	0.46^‡^	-0.02	0.15^§^	1.0

*r = Pearson's correlation coefficients; †Proportion of adherence; ‡p<0.01; §p≤0.05.

## Discussion

The findings suggest the practicability and acceptability of the Brazilian's TSQM, evidenced by the short average time of application (4.6 minutes) with 99.5% of the items answered. In the original study^3^, the mean time for application of the instrument was not reported.

Although most of the participants responded to all items, some difficulty was observed in understanding question 4, which belongs to the side effects domain, with regard to the meaning of the terms "secondary" and "collateral", used for translating the expression "side effects" in Brazilian Portuguese, as well as for understanding the terms "convenient" and "inconvenient", listed in question 11, which belong to the convenience domain. These items need revision in order to optimize the instrument for promoting better comprehension in a population with few years of study and low socioeconomic level^21^.

The results also indicated the presence of a minor ceiling effect in the effectiveness (15.8%), convenience (14.2%), global satisfaction (25.8%) domains and 90.5% in the side effects domain. It is also important to highlight that 81.1% of the participants reported no side effects. The score differences might be due to a systematic bias, such as social desirability affecting all items (the often unconscious desire to give a positive image to others by giving responses that correspond to socially admitted opinions)^22^. The social desirability bias can be changed according to the way the questionnaire is applied; it is more frequent in interview methods. Floor effect was not found in significant proportions. 

It is noteworthy that the ceiling effect occurs when a percentage of the sample scores the highest possible level of the measure, which impedes the detection of changes in situations of improvement of the health status. The floor effect occurs when a percentage of the sample scores the lowest possible level of the measure, which impedes the detection of changes in situations of worsening of the health status^10^.

The detection of these effects may indicate the impaired responsiveness of the instrument - the ability of the instrument to measure the magnitude of the change in a clinical condition over time^23^. Therefore, the Brazilian TSQM (version 1.4) can be considered potentially responsive regarding the measure of worsening of the health status, since a small floor effect was evidenced. Nevertheless, the current data suggest that there may not be a limitation regarding the detection of improvement of the health status; the ceiling effect was detected in the side effects domain (90.5%). This is expected, however, when considering the scoring technique of this domain^15^ ^-^ ^16^. Other studies reported issues regarding the distribution of the scores and ceiling and floor effects are addressed in the TSQM (version 1.4), with evidence of ceiling effect in the side effects and convenience domains^3^ ^,^ ^22^. 

Possible reasons for the occurrence of inadequate distribution of the scores were evaluated by removing respondents who infrequently reported occurrence of side effects, which resulted in a normal distribution of scores, suggesting that respondents were satisfied with drug treatment when side effects were infrequent^3^. The authors highlight that such findings should not be treated as a simple response bias, but as a result of a complex interaction between the feasibility, alternative treatments effectiveness and respondent's health status over time^3^.

In the present study, the reliability analysis was satisfactory, or close to it. The internal consistency of items, estimated by Cronbach's alpha coefficient, was demonstrated previously in the original study^3^, in which the Cronbach's alpha coefficient ranged from 0.86 up to 0.90. Other studies that applied the TSQM to assess the satisfaction with drug treatment had similar results^22^ ^,^ ^24^. 

As opposed to the previously established hypotheses, the TSQM presented no significant correlations with the measures of adherence to drug use (Table 4). The lower the Morisky score, the higher the favorability to adherence and, regarding the TSQM, the higher the score, the better the patient satisfaction, so a negative correlation is expected between these questionnaires. Nevertheless, values closer to one are still expected, not considering the sign. The lack of significant correlations between the measure of satisfaction (TSQM) and the adherence measures can be explained by the TSQM not including constructs such as medical care and treatment impact, relevant aspects pertaining to the construct of satisfaction, which can predict treatment adherence^9^. Other studies evaluated the correlation between patient satisfaction and medication adherence, and found a correlation^22^ ^,^ ^25^. However, none of them correlated TSQM (version 1.4) and the 4-item Morisky Self-Reported Measure of Medication Adherence. Instead, it was common to use the 8-item Morisky scale. It is important to highlight that the 8-item Morisky Medication Adherence scale is not available in Brazilian Portuguese. 

Adherence consists of a complex measure for which there is no gold standard. In the present study, adherence was assessed by two instruments as recommended elsewhere^26^, in order to respect its aspects and maximize its accuracy. Even though the Morisky Self-Reported Measure of Medication Adherence is widely used, its reliability varies among different samples^19^. Future studies could consider objective measures of adherence (such as direct observation of medication intake or use of biological markers), and the Morisky self-reported measure would increase the accuracy of the adherence measure. 

In the future, health care providers in the field of chronic diseases have to focus their attention on the aging population, their prevalence will be much more frequent, as well as the necessity of continuous and persistent medication treatment^3^. It is known that patient satisfaction influences the health behavior and would be crucial in this process of treatment in patients with chronic diseases, mainly in patients with CHD, in which the treatment adherence reduces the number of ischemic events and improves quality of life. Nurses as health educators would be pivotal in educating patients about the self-management of diseases, thus increasing the patient satisfaction and achieving high adherence rates^25^.

Further studies are recommended in order to deeply evaluate the psychometric performance of the TSQM, aimed at confirming its validity, sensitivity and the structure of the factors. The results of this study present relevant implications for nurses and other health professionals, since they support a stronger evaluation of a questionnaire that can be used to assess the effectiveness of interventions to improve patients' satisfaction with their drug treatment.

## Limitations

One of the limitations were the interview technique in which the instruments were applied, including TSQM (version 1.4), although the application of the TSQM as a self-reported measure is recommended, and interviews can lead to social desirability. Even without testing the cognitive function or levels of comprehension, the low levels of study and family income motivated this approach, as the majority of the Brazilian population was considered. 

As for the construct validity, it is important to highlight that there is no gold standard for an adherence measurement and, on top of that, adherence can be deceptive - in order to achieve more precise results, we have applied two questionnaires to measure this construct.

## Conclusion

The results of this study indicate that the Brazilian TSQM (version 1.4) is a questionnaire with easy application, with evidence of acceptability and potential sensitivity in detecting worsening regarding patients' satisfaction with their medication, evidenced by the low floor effect, as well as the limitation in detecting improvement regarding patient satisfaction by the finding of a substantial ceiling effect in the side effects domain. Construct validity was weakly supported, since small significant correlations were observed with the general measure of satisfaction and adherence.

In the era of patient safety, nurses play a pivotal role as they not only provide direct health care to patients, but also regarding their health education and behavior. Patients who perceive their medication to be ineffective, laden with side effects, or too inconvenient to use are less likely to take their medication as prescribed. Thus, dissatisfaction with medication may affect the effectiveness of treatment and result in treatment failure. Nurses may conduct regular evaluation of patient satisfaction by applying the TSQM, which aids the health team to monitor individuals whose current experiences may increase the risk of low medication adherence.

Further studies are recommended in order to confirm the structure of the factors (factor analysis) of the Brazilian version of the TSQM and its validity, as well as to investigate its sensitivity and responsiveness.
